# Scrofuloderma with disseminated tuberculosis in an Ethiopian child: a case report

**DOI:** 10.1186/s13256-018-1927-1

**Published:** 2018-12-17

**Authors:** Henok Tadele

**Affiliations:** Department of Pediatrics and Child Health, College of Medicine and Health Sciences, Hawassa University, Hawassa, Ethiopia

**Keywords:** Cutaneous tuberculosis, Scrofuloderma, Xpert MTB/RIF, Child, Ethiopia

## Abstract

**Background:**

Cutaneous tuberculosis represents only 1–2% of extrapulmonary forms of tuberculosis. Scrofuloderma is an endogenous form of cutaneous tuberculosis and can present as isolated or coexist with pulmonary and disseminated forms of tuberculosis. Pathologically confirmed scrofuloderma coexisting with disseminated tuberculosis with a good treatment response is presented and discussed.

**Case presentation:**

A 12-year-old African Ethiopian girl presented with bilateral neck swelling with purulent discharge and skin ulceration of 3 months’ duration. Dry cough, low-grade fever, decreased appetite, drenching night sweats, global throbbing headache, and a significant amount of weight loss were also reported. Biopsy of the skin identified scrofuloderma, and *Mycobacterium tuberculosis* was also identified by Xpert MTB/RIF assay. Cerebrospinal fluid analysis and brain computed tomographic scans showed tuberculous meningitis and tuberculoma. Antituberculosis therapy with rifampicin, isoniazid, pyrazinamide, and ethambutol; prednisolone; pyridoxine; and wound care were provided. The patient was discharged for outpatient directly observed antituberculosis therapy in a nearby health center after acute complications were treated and once the skin lesion had started to dry or heal.

**Conclusions:**

Cutaneous tuberculosis should be considered in a child presenting with a skin lesion or discharge. Cutaneous tuberculosis cases should be investigated for coexisting pulmonary and extrapulmonary forms of tuberculosis. Histopathologic diagnosis should be considered to rule out other skin pathologies and also to prevent delay in treatment. Better tuberculosis prevention strategies, including vaccination scale-up, are warranted.

## Introduction

Tuberculosis (TB) has remained a global public health problem and has diverse pulmonary and extrapulmonary presentations [1]. Cutaneous tuberculosis (CTB) represents only 1–2% of extrapulmonary forms of TB [2]. CTB is classified into several variants, and scrofuloderma is a form of endogenous CTB [2, 3]. Scrofula, tuberculous lymphadenitis of the neck, is named for the likeness of the cluster of nodes to piglets feeding from a sow [4].

Scrofuloderma affects people of all ages. However, children, teenagers, and elders are highly affected, owing to immunologic failure to contain mycobacterial infection [5]. Scrofuloderma can present in an isolated form or coexist with pulmonary and disseminated forms of TB [2]. It presents as erythematous nodules that fistulize and discharge caseous and purulent material [6]. Pathologic examinations reveal abscesses, necrosis, and tuberculoid granulomas [7, 8]. In this report, pathologically confirmed scrofuloderma coexisting with disseminated TB in an unvaccinated teenager with good clinical response is presented and discussed. CTB is a rarely reported form of TB, and this case report highlights the need for CTB consideration and a confirmatory pathologic test in childhood skin lesions. This case emphasizes the need for workup to detect coexisting extrapulmonary TB.

## Case presentation

A 12-year-old African Ethiopian girl presented with bilateral neck swelling with purulent discharge and skin ulceration of 3 months’ duration. She had dry cough, low-grade fever, decreased appetite, and drenching night sweats, and she had lost a significant amount of weight. She had a global throbbing type of headache with occasional projectile vomiting of ingested matter starting 6 days before her visit. She had never been vaccinated and had no known contact with any person diagnosed with TB or with any chronic cougher. She had not received any treatment for the complaints prior to her current visit. She had no known medical problem, and her family and psychosocial history were unremarkable.

**Fig. 1:**
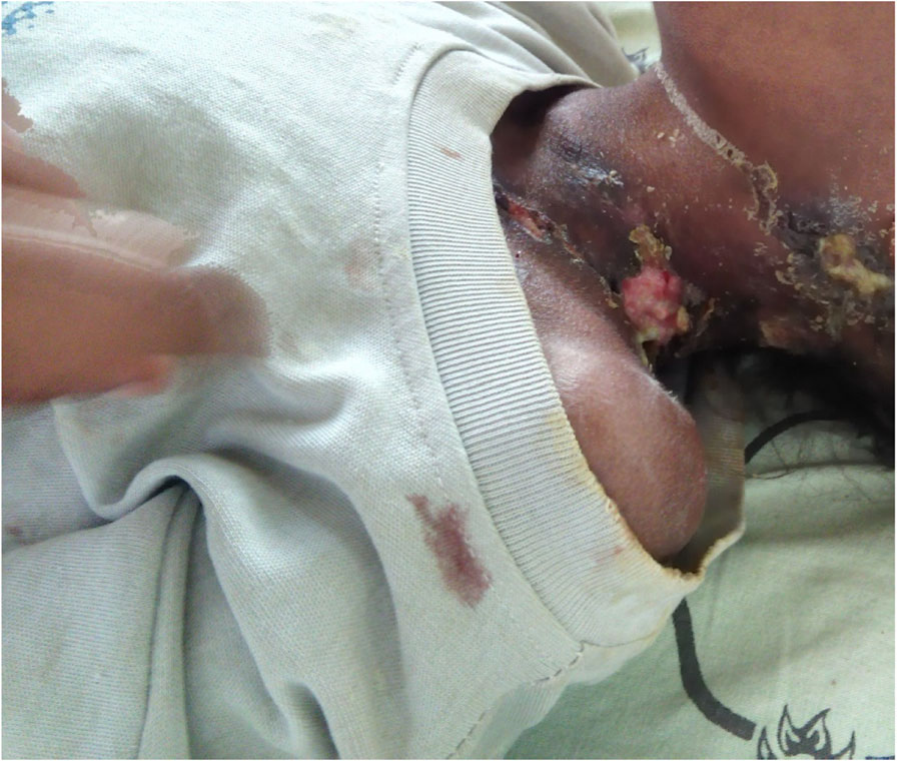
Multiple left cervical lymphadenopathy with purulent drainage and skin ulceration

**Fig. 2:**
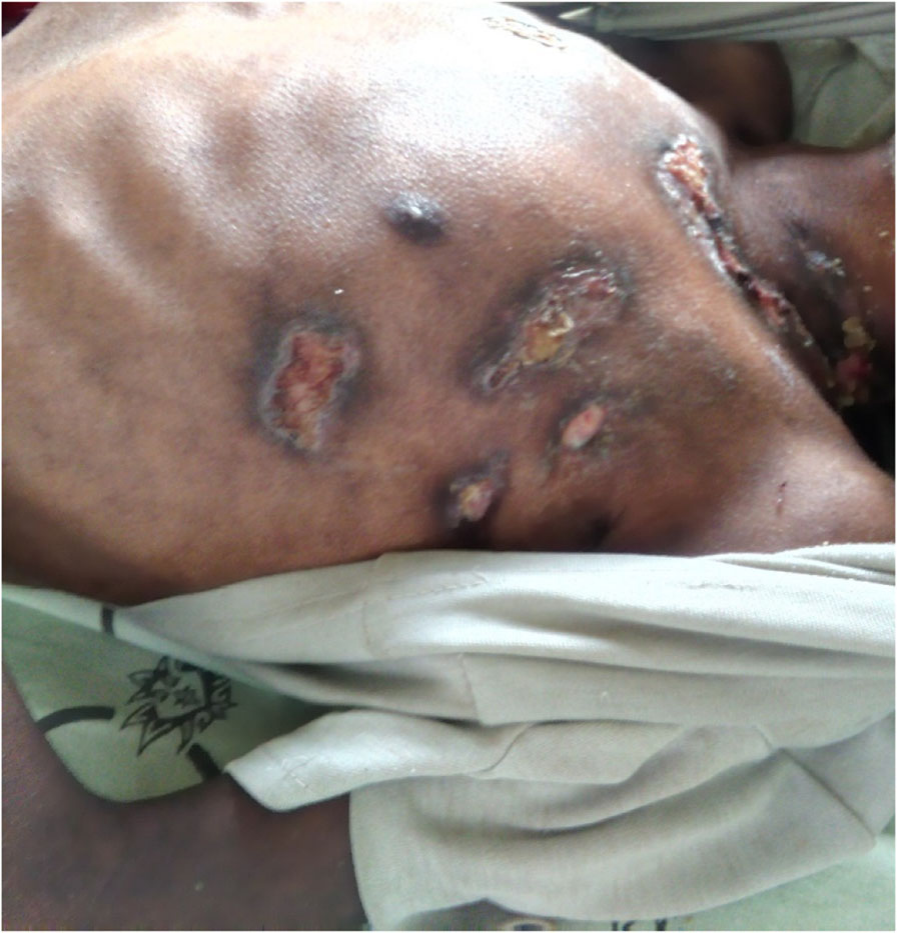
Ulcerated skin lesions over the anterior trunk with purulent discharge

Her physical examination revealed a conscious, emaciated, and wasted child. Her admission vital signs were pulse rate 80 beats/min, respiratory rate 20 breaths/min, blood pressure 90/60 mmHg, and axillary temperature 37.3 °C. Matted bilateral anterior cervical and postauricular lymphadenopathy with pus-draining sinus was noted. Crepitation over the anterior and posterior lower right chest, multiple skin ulcers with purulent drainage over the left lateral neck, anterior left chest, and left axilla were documented (*see* Figs. 1 and 2). Nuchal rigidity was positive, but no neurologic deficit was present.

**Fig. 3:**
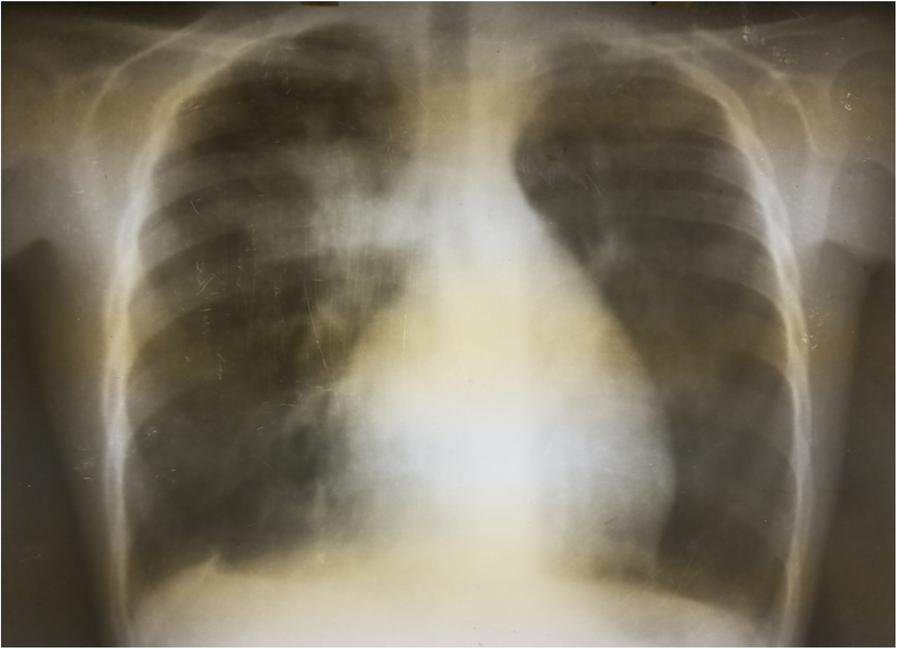
Chest x-ray of the patient

**Fig. 4:**
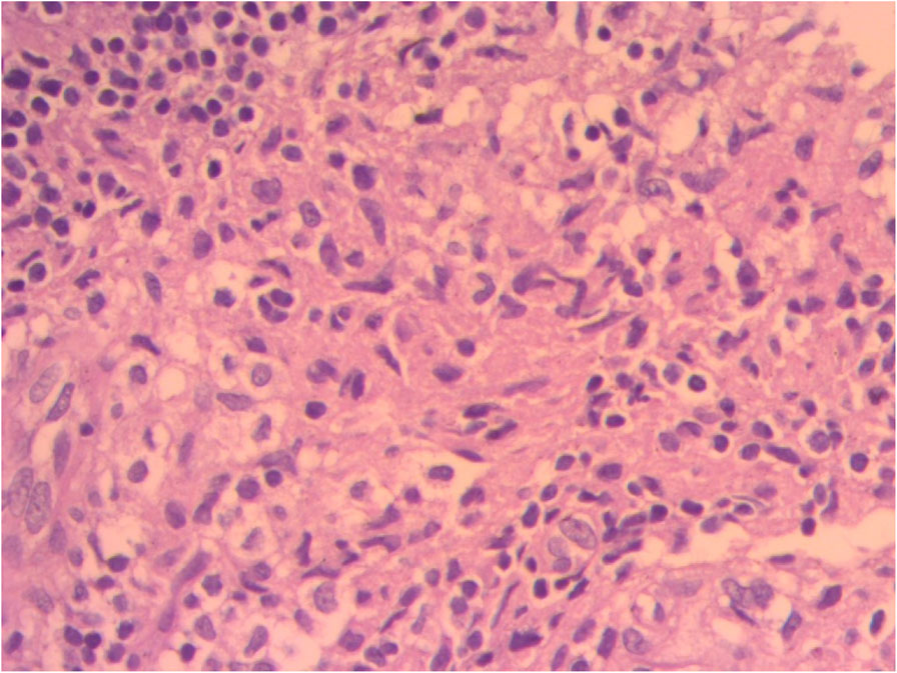
Histopathologic smear of the patient

**Fig. 5:**
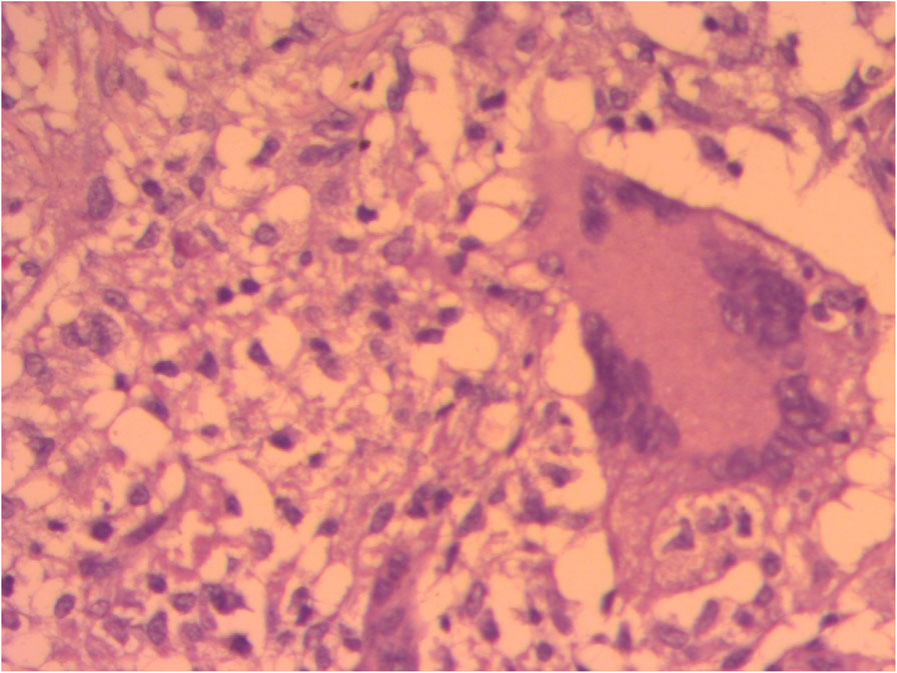
Histopathologic smear of the patient

**Fig. 6:**
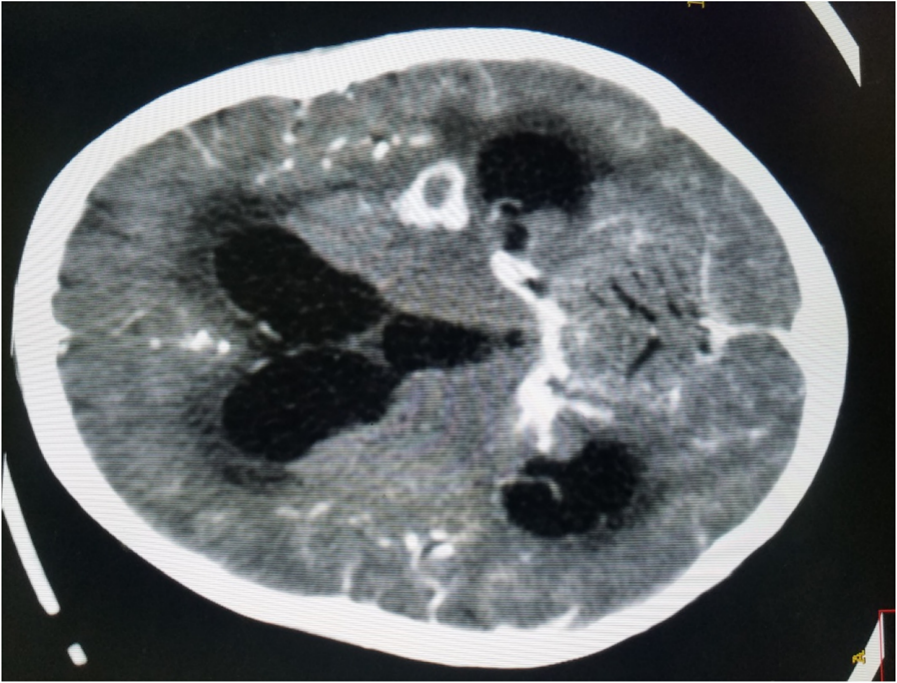
Brain computed tomographic scan showing ring-enhancing lesions with intense basal cisterns exudate

**Fig. 7:**
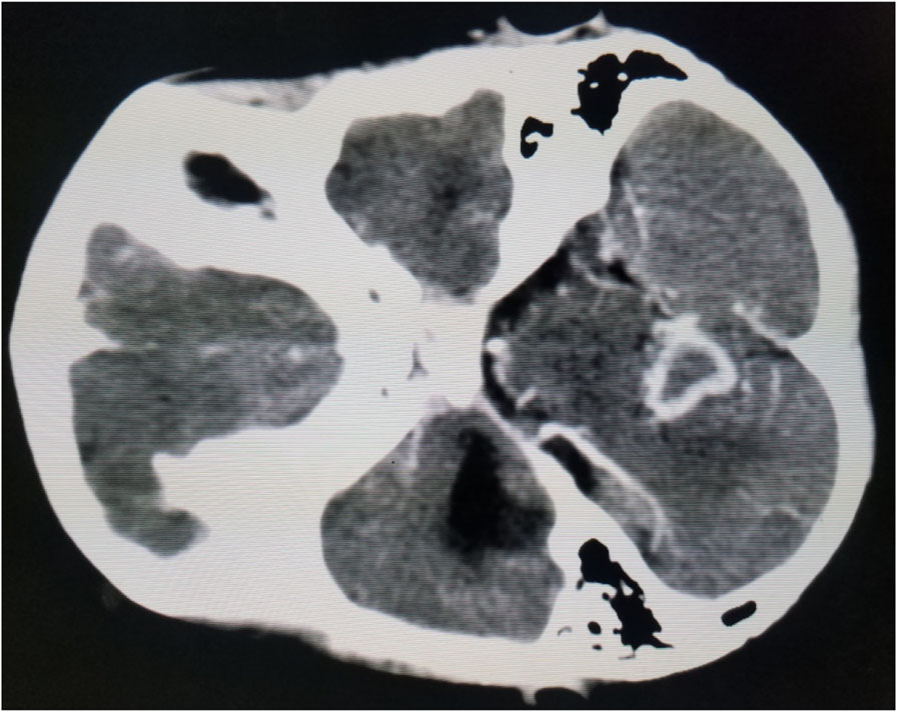
Brain computed tomographic scan showing ring enhancing lesion with perilesional edema

**Fig. 8:**
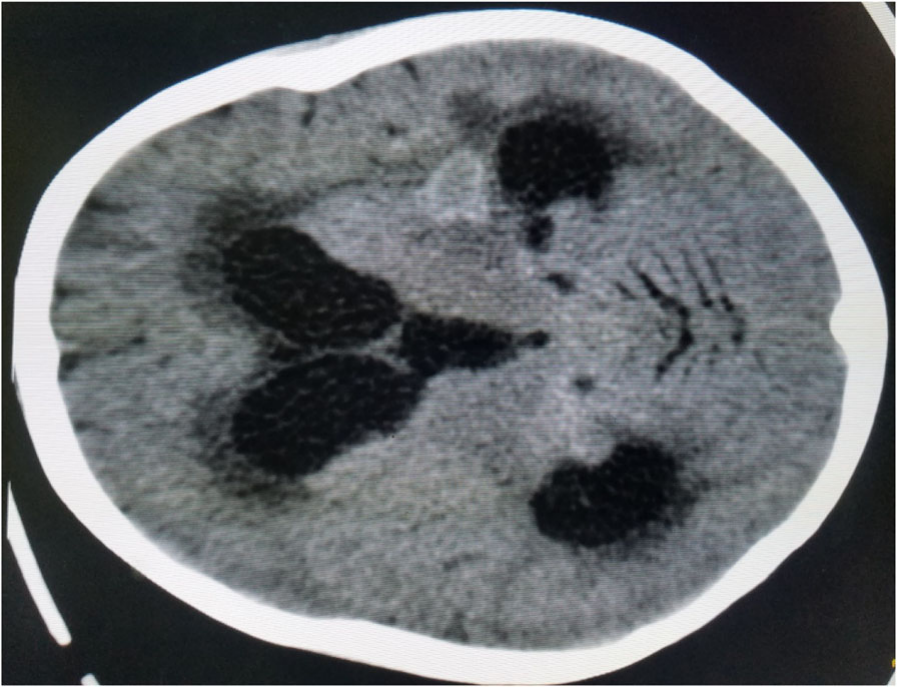
Brain computed tomographic scan showing dilated ventricles with solid tuberculoma

The results of complete blood count, urinalysis, and biochemical analysis were normal, except for mild anemia (*see* Table 1). The result of an antibody test for human immunodeficiency virus was negative. The patient’s erythrocyte sedimentation rate was 65 mm/hr in the first hour. Cerebrospinal fluid analysis showed 105 cells with 65 lymphocytes, and no organism was detected on Gram stain and acid-fast bacilli stain. Right upper and middle lobe ill-defined air space opacity was noted on a chest x-ray (*see* Fig. 3). Discharge analysis from the skin revealed gram-positive diplococci in chain, and *Mycobacterium tuberculosis* was detected using the Xpert MTB/RIF assay (Cepheid, Sunnyvale, CA, USA). Fine-needle aspiration cytology showed only caseous necrosis and no granuloma, and smears from ulcerated lesions showed mixed inflammatory cells, predominantly polymorphs. Tuberculous scrofuloderma was diagnosed on the basis of skin biopsy with a section showing mature squamous cell-lined skin tissue composed of epithelioid cell granuloma, multinucleated giant cells, caseous necrosis, and mixed inflammatory cells (*see* Figs. 4 and 5). Brain computed tomographic scans showed multiple precontrast hyperdense randomly distributed supra- and infratentorial lesions with mild perilesional edema. Rim enhancement on postcontrast images (caseating granulomas) on some lesions and a solid pattern of enhancement (noncaseating granulomas) were evident on other lesions. Dense basal cistern exudate seen on precontrast images with avid postcontrast basal leptomeningeal enhancement was observed. Severe bilateral lateral third- and fourth-ventricle dilation was noted (*see* Figs. 6, 7 and 8, Table 1). A tuberculin skin test (TST) could not be done, owing to unavailability of the service.

**Table 1:** Investigations and findings

Serial number	Investigations	Results or findings
1	Complete blood count	WBC 13.9 × 10^3^ Red blood cell count 4.4 × 10^3^ Platelet count 278 × 10^3^ Hematocrit 10 g/dl
2	Urinalysis	pH 6 Specific gravity 1.020 Negative for ketone and glucose WBC 2–5/HPF Negative for chemical tests (leukocyte esterase, nitrite, urobilinogen, protein)
3	Liver function tests	AST/SGOT (aspartate transaminases) 92U/L SGPT/ALT (alanine aminotransferase) 40U/L ALP (alkaline phosphatase) 100IU/L
4	Renal function test	Creatinine 0.5mg/dl
5	HIV	Negative antibody test
4	Erythrocyte sedimentation rate in first hour	65 mm/hr
2	Cerebrospinal fluid analysis	105 cells (65 lymphocytes) No organism on Gram stain and acid-fast bacilli stain
3	Chest x-ray	Right upper and middle lobe ill-defined airspace opacity
4	Skin discharge analysis	Gram-positive diplococci in chain on Gram stain, *Mycobacterium tuberculosis* on Xpert MTB/RIF assay
5	Fine-needle aspiration cytology	Only caseous necrosis, no granuloma, and smears from ulcerated lesions show mixed inflammatory cells, predominantly polymorphs
6	Skin biopsy	Tuberculous scrofuloderma: with section showing mature squamous cell-lined skin tissue composed of epithelioid cell granuloma, multinucleated giant cells, caseous necrosis, and mixed inflammatory cells
7	Brain computed tomography	Multiple precontrast hyperdense randomly distributed supra- and infratentorial lesions with mild perilesional edema. Rim enhancement on postcontrast image (caseating granulomas) on some lesions and solid pattern of enhancement (noncaseating granulomas) evident on others. Dense basal cistern exudate seen on precontrast images with avid postcontrast basal leptomeningeal enhancement. Severe bilateral lateral, third- and fourth-ventricle dilation was noted.

*Abbreviations: HIV* Human immunodeficiency virus, *HPF* High-power field, *Xpert MTB/RIF* Automated real-time nucleic acid amplification technology used for rapid and simultaneous detection of tuberculosis and rifampicin resistance, *WBC* White blood cell

Routine care and medical treatment was provided as per World Health Organization (WHO) guidelines for severe acute malnutrition. Antituberculosis therapy with rifampicin, isoniazid, pyrazinamide, and ethambutol as per weight (four tabs per day), prednisolone 2 mg/kg/day, and pyridoxine 25 mg/day was started. Wound care was also given. After a 1-month hospital stay, the patient was discharged for outpatient directly observed antituberculosis therapy in a nearby health center after acute complications were treated and once the skin lesion had started to dry or heal. She received antituberculosis therapy for 12 months. No drug-related side effects were seen or reported during admission or follow-up. Follow-up visits (including 6 months after completion of therapy) demonstrated a patient who had recovered well with no major neurologic problems and with healed skin lesions.

## Discussion

This case report presents a rare form of TB, CTB. Our patient was an unvaccinated 12-year-old African Ethiopian girl who presented with purulent discharge and skin ulceration of 3 months’ duration. She had dry cough, low-grade fever, decreased appetite, drenching night sweats, and global throbbing headache, and she had lost a significant amount of weight. Pathologic tests confirmed scrofuloderma coexisting with disseminated TB. She was managed, and she responded well to the treatment. This case report uniquely presents CTB coexisting with extrapulmonary TB and calls for better clinical assessments and workup for coexisting forms of TB. It also stresses the need for a confirmatory pathologic skin test to avoid a delay in treatment initiation.

CTB is a rare form of TB [2], and Ethiopia has one of the highest TB burdens worldwide [1]. An Ethiopian study of adult patients documented CTB to be the second most common histopathologically diagnosed skin disorder [9]. Scrofuloderma is the commonest form of CTB in children and almost always is of the endogenous type [10, 11]. Children with CTB tend to have a disseminated form of TB, as in our patient [12].

CTB diagnosis is a challenge, owing to difficulty in recovering the bacilli. Generally, diagnostic methods have a low sensitivity and specificity for CTB compared with the pulmonary form of TB. For suspected CTB, it is advised to do a TST, obtain a chest x-ray, and perform histopathologic examination and culture for acid-fast bacilli as well as PCR of skin sample and blood [13].

The TST result tends to be positive in CTB (33–96% of cases), creating difficulty in differentiating whether positivity is for true mycobacterial infection or nontuberculous mycobacterial infection and/or related to bacille Calmette-Guérin vaccination. This is a common problem in endemic TB settings such as Ethiopia. Even then, it is not a routine practice in Ethiopian settings to do a TST for TB screening, and hence it was not done in our patient [14, 15]. Extrapulmonary TB diagnosis, including CTB, using the Xpert MTB/RIF assay is recommended by the WHO [14]. The Xpert MTB/RIF test of skin discharge revealed *M. tuberculosis* in our patient.

Histologic diagnosis of CTB is based on pathologic findings of lymphocytes, epithelioid histiocytes, giant cells, necrosis, and granuloma. Histology assists in differentiating other skin pathologies that mimic TB [13, 15, 16]. Our patient’s skin histologic examination results are depicted in the figures and support the diagnosis of scrofuloderma.

Treatment of CTB is similar to pulmonary TB and will be prolonged for coexisting extrapulmonary forms of TB. Recommended treatment includes a 2-month four-drug regimen (isoniazid, rifampicin, pyrazinamide, and ethambutol), followed by two-drug therapy (isoniazid and rifampicin). The duration of the two-drug regimen is decided on the basis of the patient’s clinical condition. Pyridoxine is recommended for malnourished children to alleviate the neurologic side effects of isoniazid [1, 17, 18]. Our patient had a central nervous system and pulmonary form of TB coexisting with CTB. Recommended treatment with four antituberculosis drugs, pyridoxine, and a steroid (prednisolone) was initiated. Severe acute malnutrition was managed as per WHO guidelines [19].

Early detection of TB through patient-initiated and screening pathways needs to be reinforced. Early detection or diagnosis, especially among high-risk groups such as children will help to alleviate death and disability related to TB [20]. Our patient had symptoms of TB for months, and early TB detection programs or systems could have prevented the severe and disfiguring form of TB in our patient.

## Conclusions

This report presents a case of a patient with a rare form of extrapulmonary TB. CTB should be considered in a child presenting with a skin lesion or discharge. CTB cases should be investigated for coexisting pulmonary and extrapulmonary forms of TB. A histopathologic diagnosis should be considered to rule out other skin pathologies and also to prevent delay in treatment. TB prevention strategies, including vaccination, need to be strengthened.

## Abbreviations

CTB: Cutaneous tuberculosis
TB: Tuberculosis
TST: Tuberculin skin test
WHO: World Health Organization
Xpert MTB/RIF: Automated real-time nucleic acid amplification technology used for rapid and simultaneous detection of tuberculosis and rifampicin resistance
